# Combination Therapy Should Be Reserved as Second-Line Treatment of Onychomycosis: A Systematic Review of Onychomycosis Clinical Trials

**DOI:** 10.3390/jof8030279

**Published:** 2022-03-09

**Authors:** Julianne M. Falotico, Rebecca Lapides, Shari R. Lipner

**Affiliations:** 1 Renaissance School of Medicine at Stony Brook University, Stony Brook, NY 11794, USA; julianne.falotico@stonybrookmedicine.edu; 2 Robert Larner, M.D. College of Medicine at the University of Vermont, Burlington, VT 05405, USA; rebecca.lapides@med.uvm.edu; 3 Weill Cornell Medicine, Department of Dermatology, New York, NY 10021, USA

**Keywords:** onychomycosis, nail disease, fungal nail infection, randomized controlled trial, clinical trial, combination therapy, monotherapy

## Abstract

Onychomycosis is the most common nail disease encountered in clinical practice. Its importance extends well beyond aesthetics, often causing pain, difficulty with ambulation and performing daily activities, and impairing quality of life. Many patients fail to achieve cure with antifungal monotherapy and recurrences are common. Combination therapy has therefore gained considerable interest, given the potential for drug synergy and prevention of antifungal resistance, but it has not been well studied. A systematic review of onychomycosis medication only, as well as medication and procedural (laser, debridement, photodynamic therapy), clinical or randomized controlled trials evaluating combination vs. monotherapies was performed. After exclusions, 30 studies were included in the final analysis. There were conflicting results for medication-only trials, with some showing significant benefit of combination therapy over monotherapy, however, trials were not robustly designed and lacked sufficient follow-up. Procedural studies also lacked long-term follow-up, and failed to demonstrate efficacy in some severe onychomycosis cases. Considering the high cure rates demonstrated in pivotal antifungal monotherapy trials, and conflicting results, costs, and safety concerns associated with combination therapy, we recommend that combination therapy be reserved as second-line treatment options in patients with poor prognostic factors or for those who failed monotherapy for onychomycosis.

## 1. Introduction

Onychomycosis is a fungal nail infection due to dermatophytes, nondermatophytes, and yeast [1], clinically presenting with nail plate onycholysis, thickening, and subungual hyperkeratosis [2], with significant physical, aesthetic, and psycho-social consequences. Currently, oral terbinafine, itraconazole, and griseofulvin, as well as topical ciclopirox, efinaconazole, and tavaborole, are United States (US) Food and Drug Administration (FDA) approved for onychomycosis treatment, and oral fluconazole is often used off-label [3,4]. In a systematic review and meta-analysis of 26 randomized controlled trials (RCT) investigating systemic monotherapy for toenail onychomycosis [5], there was a significantly greater odds ratio (OR) of achieving mycological cure for all monotherapy treatments vs. placebo. Onychomycosis treatment is challenging, and is individualized based on disease severity, co-morbidities, and infecting organism(s), with consideration of associated medication adverse events, drug–drug interactions, and cost [1,6]. Some disadvantages of onychomycosis monotherapy include potential antifungal resistance, and difficulty of achieving high concentrations of biologically effective drug in affected nails, particularly in severe cases of onychomycosis [7]. In a five-year, blinded follow-up study of patients achieving mycological cure at 12 months following oral terbinafine or itraconazole monotherapy treatment, 23% and 53% of patients, respectively, experienced mycological relapse or reinfection [8]. Therefore, there is a need for studies with long term follow-ups to track recurrences after complete cures to optimize treatments regimens and prevent recurrences.

Combinations of oral medications, topical medications, and devices, have been considered in cases where there are expected poor responses to monotherapy, greater than 50–60% nail involvement, or more than three affected nails [4,9,10]. Parallel, or simultaneous, combination therapy is recommended in patients likely to fail therapy (i.e., with underlying comorbidities such as diabetes), while sequential therapy is recommended in patients with poor responses to initial treatment [11]. It is theorized that combination therapy allows for antimicrobial synergy, broader antifungal coverage with increased fungicidal activity, and decreased resistance [12], as well as improved clinical cures when using drugs with different mechanisms of action or administration routes [13]. However, research on combination antifungal therapy for onychomycosis is sparse and the most recent reviews date from 1999–2006 [7,11,12,13,14,15,16]. In this systematic review, we examine clinical trials comparing combination vs. monotherapy for onychomycosis treatment, to guide clinical management.

## 2. Materials and Methods

**Figure 1:**
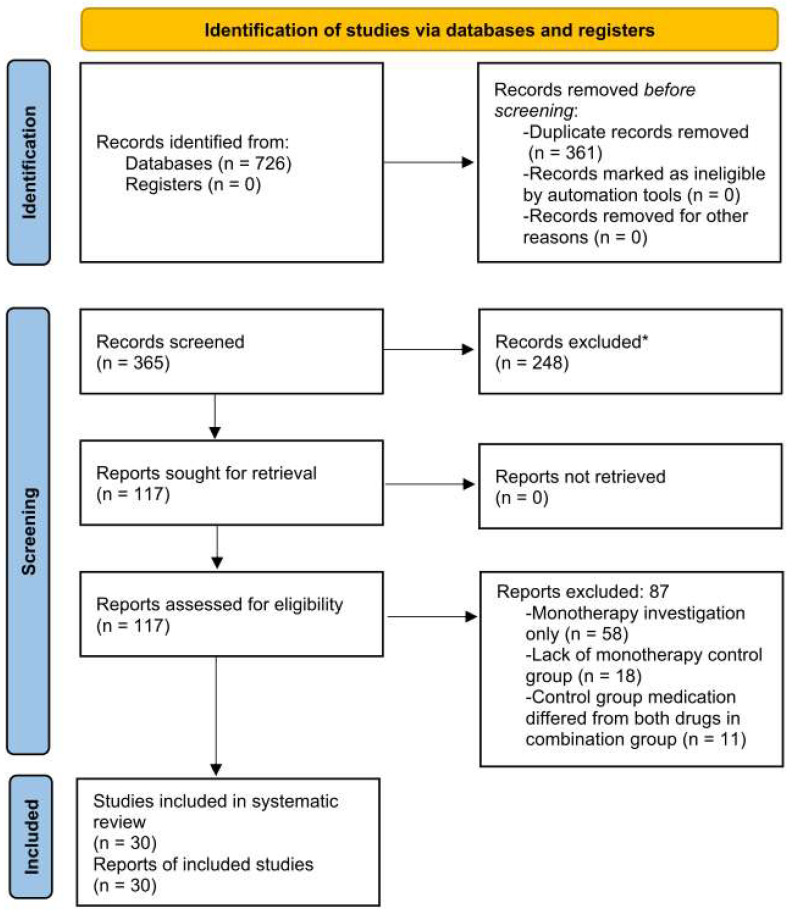
PRISMA flow diagram for systematic review procedure. * Records were manually screened with no automation tools used.

The objective of this systematic review was to examine combination therapy for onychomycosis treatment, and is reported in accordance with PRISMA guidelines [17]. This review was not registered and a protocol was not prepared. PubMed, Scopus, and Web of Science databases were searched for articles on onychomycosis combination therapy on 1 July 2021, for all peer-reviewed, English-language, human subject onychomycosis clinical and RCTs with no date ranges, and using search terms “onychomycosis treatment”, “onychomycosis therapy”, and “onychomycosis combination therapy”. Articles were independently screened by two authors (R.L. and S.R.L.) based on abstracts. Both authors then independently reviewed full-text articles for eligibility and extracted data for eligible studies. Inclusion required investigation of a combination therapy versus monotherapy for onychomycosis treatment, and mycological confirmation with microscopy, culture, or another validated laboratory-based testing method prior to treatment initiation. Duplicate, non-English, non-randomized, non-clinical trials, and studies investigating monotherapy treatment regimens, diagnoses other than onychomycosis (i.e., tinea pedis), lacking monotherapy control groups, or control group medications that differed from both drugs in the combination group were excluded. Outcomes, including number of subjects, treatment protocol, treatment success rate, and adverse effects, were extracted from each study. Data that was not available was stated (N/A). The PRISMA flow diagram (Figure 1) provides additional information regarding the systematic search.

## 3. Results

There was a total of 726 studies from the initial search, with 30 clinical trials (2531 participants) meeting inclusion criteria and included in the final analysis (Figure 1). Half (15/30) of the studies investigated medications only (Table 1) and half studied procedures (debridement, photodynamic therapy, lasers) in combination with medication (Table 2).

Of the 15 medication-only studies (Table 1), the average number of subjects was 139.1 [standard deviation (SD): 135.5; range: 10–595], with average treatment duration of 31.0 weeks (SD: 21.4; range 4–65). Terbinafine (8/15, 53.3%) and amorolfine (6/15, 40.0%) were the most commonly studied oral and topical medications, respectively. Most studies (11/15, 73.3%) investigated an oral medication in combination with a topical medication, with oral terbinafine and topical amorolfine (4/11, 36.4%), and oral terbinafine and topical ciclopirox (3/11, 27.3%) being most common, with more than half (7/11, 63.6%) designed with the topical medication administered for longer than the oral medication. Common endpoints assessed were mycological cure rates in 13 (86.7%), complete cure rates in 10 (66.7%), and clinical cure rates in 6 (40.0%) studies, with 5 studies (33.3%) reporting all 3 cure rates.

Significant clinical benefit of medication combination therapy vs. monotherapy was observed in 60% (9/15) of studies (Table 1). Studies investigating oral terbinafine and topical amorolfine reported significantly greater mycological cure rates at 3 months (94.2% vs. 59.7%; *p* < 0.001) [22], and complete [72.3% vs. 37.5%; 95% confidence interval (CI): 57.4–84.4 vs. 23.9–52.6) [20], (59.2% vs. 45.0%; *p* = 0.03) [22], and clinical cure rates (74% vs. 42%; 95% CI: 60–86 vs. 28–57) [21] at 18 months in the combination vs. oral terbinafine monotherapy groups. Studies examining oral griseofulvin combined with topical tioconazole or bifonazole showed significantly higher complete cure rates at 12 months (69% vs. 41%; *p* < 0.005) [18], and significantly higher mycological cure rates (93% vs. 66%, *p* < 0.01) and lower relapse rates (7% vs. 20%, *p* < 0.01) at 4 months [19] compared to oral griseofulvin monotherapy treatment. Combination of topical therapies only (bifonazole and urea) resulted in significantly greater complete (50.7% vs. 40.9%; *p* = 0.0260) and mycological (61.5% vs. 49.1%, *p* = 0.0033) cure rates at 3 months compared to urea monotherapy, but this result was not sustained at 6 months [26]. Combinations of topical fluconazole and urea showed higher mycological cure (82.8% vs. 62.6%) and clinical improvement (77.1% vs. 68.0%) rates than fluconazole alone at 6 months, however *p*-values were not reported [27].

Conflicting results were found in studies combining oral itraconazole and topical amorolfine vs. itraconazole monotherapy, with one study reporting significantly greater mycological (≥90% vs. <69%; *p* < 0.001) and complete cure rates (83.7–93.9% vs. 68.8%; *p* = 0.011) at 24 weeks [28], and another reporting no significant differences at 3 months [29]. One study on oral terbinafine and topical ciclopirox combination therapy vs. oral terbinafine monotherapy found significantly greater mycological cure rates (88.2% vs. 64.7%; *p* = 0.043) and markedly improved or cured target toenail (82.4% vs. 58.8%; *p* = 0.004) at 9 months [23], while others reported no significant differences at 48 weeks [24] or 9 months [25]. One study investigating oral therapies only (itraconazole and terbinafine) found significantly higher mycological (66.7% vs. 46.3%; *p* = 0.007), complete (48.1% vs. 30.5%; *p* = 0.03), and effective therapy (60.5% vs. 43.2%; *p* = 0.02) rates at 72 weeks vs. oral terbinafine monotherapy [30], while another found no significant differences in recurrence rates at 48 weeks [31]. Combinations of oral itraconazole or griseofulvin and topical isoconazole or urea showed no clinical benefit of combination therapy over monotherapy [32].

In seven medication-based studies that reported adverse events, 57.1% reported greater adverse events in monotherapy groups compared to combination groups. Cost (treatment cost per cured patient or cost per cure ratio) for oral terbinafine and topical amorolfine vs. oral terbinafine [22] and oral itraconazole and topical amorolfine vs. oral itraconazole [28,29] was lower for combination therapy than monotherapy.

Of the 15 studies investigating medications combined with procedures (Table 2), the average number of subjects was 114.2 (SD: 160.82; range: 9–504) with an average treatment duration of 21.4 weeks (SD: 13.4; range: 3–52). The majority of studies (10/15, 66.7%) utilized laser therapy, with laser and topical therapy (7/10, 70%) more common than oral therapy (3/10, 30%), and 1064-nm Nd:YAG being the most commonly used laser (6/10, 60%). The most common combination overall was laser and topical amorolfine (3/15, 20%), and the most common medications were oral terbinafine (4/15, 26.7%) and topical amorolfine (3/15, 20%). Common endpoints assessed were mycological cure rates in 11 (73.3%), clinical cure rates in seven (46.7%), and complete cure rates in two (13.3%) studies, with only one study (6.7%) reporting all three cure rates.

Significant clinical benefit of procedural and medication combination therapy vs. monotherapy was observed in almost all (14/15, 93.3%) studies (Table 2). Studies investigating laser therapy and topical amorolfine reported significantly greater mycological cure rates at 24 weeks (75 vs. 20; *p* = 0.001) [33] and 3 months (65% vs. 35%; *p* = 0.05) [34] in the combination vs. topical amorolfine and laser monotherapy groups, respectively. Similar improvements in mycological cure rates at 24 weeks (66.67–100% vs. 35.29–61.54%, *p* < 0.05), as well as decreases in onychomycosis severity index (OSI) scores (2–8 vs. 1–4; *p* < 0.002) were reported in another study [35]; however, significant differences were only observed in patients with mild and moderate onychomycosis, with no significant improvements in patients with severe onychomycosis treated with combination therapy for either metric (*p* > 0.05, both). Laser and topical efinaconazole combination resulted in significantly greater improvement in the Scoring Clinical Index for Onychomycosis (SCIO) index at 36, 48 (both *p* = 0.04), and 52 weeks (*p* = 0.02) compared to topical efinaconazole monotherapy, with no significant difference in mycological cure rates [36]. Laser and topical tioconazole had significantly higher clinical cure, patient satisfaction, negative potassium hydroxide, and negative fungal culture (55%, 60%, 80%, 70%, respectively) rates than laser (30%, 40%, 55%, 30%, respectively) or topical tioconazole (25%, 30%, 55%, 30%, respectively) monotherapies (*p* < 0.05, all) [39]. Laser and topical luliconazole resulted in significantly greater mycological cure rates 3 months after the last treatment (69.6% vs. 57.4%; *p* = 0.006) and clinical cure rates at 3 (69.6% vs. 50.9%; *p* = 0.004) and 6 months (73.0% vs. 52.8%; *p* = 0.002) than laser monotherapy [40]. Laser and topical naftifine hydrochloride spray showed significant improvements in mycological (22.5% vs. 4.5%) and clinical cure (40.8% vs. 7.5%) at 24 weeks, however laser monotherapy also showed significant benefit over topical monotherapy for both clinical and mycological cure rates (*p* < 0.005, all) [42].

In studies assessing laser and oral itraconazole combination therapy, efficacy rate (cure (new clear nail growth with less than 5% nail dystrophy) plus significant efficacy (60% new clear nail growth) rates) were significantly higher at 8 (20% vs. 13%) and 24 weeks (21% vs. 11%) (*p* < 0.05, both) compared to laser monotherapy, only for patients with severe onychomycosis, with no significant differences across groups in patients with mild/moderate onychomycosis (*p* > 0.05) [37]. In another study, overall clinical cure rate was significantly greater (*p* = 0.001) and mean OSI score after treatment significantly lower (*p* < 0.01) in laser and oral itraconazole combination group vs. oral itraconazole monotherapy, however mycological responses did not significantly differ [38]. Laser and oral terbinafine combination therapy resulted in significantly greater mycological and clinical cure rates at 4, 8, 12, 16, and 24 weeks vs. both laser and terbinafine monotherapies (*p* < 0.05, all) [41].

Studies examining debridement and oral terbinafine vs. terbinafine monotherapy reported significant improvements in symptom frequency (*p* = 0.0395) and treatment satisfaction (*p* = 0.0077) based on a validated onychomycosis-specific patient-reported outcomes questionnaire [45], and significantly greater clinical cure rates (59.8% vs. 51.4%; *p* = 0.023) [44], however there were no differences in mycological or complete cure rates (*p* > 0.05). Debridement with topical ciclopirox resulted in significantly greater mycological cure rates than debridement alone (76.74% vs. 0%; *p* < 0.05) [46]. Combination of nail drilling plus oral and topical terbinafine resulted in significantly greater mean percent clear nail at 16 (63.75% vs. 31.36%; *p* = 0.028) and 22 weeks (59.38% vs. 23.81%; *p* = 0.005) vs. topical terbinafine monotherapy, while nail drilling and topical terbinafine combination therapy resulted in significant improvement in mean percent clear nail at 22 weeks only (52.39% vs. 23.81%; *p* = 0.014) [47]. Mycological cure rates did not differ significantly amongst any groups. Methyl aminolevulinate photodynamic therapy and topical urea combination therapy showed no significant differences in mycological (*p* = 0.178) or complete (*p* = 0.23) cure rates vs. urea monotherapy [43].

Adverse events were reported in seven studies, with almost half (3/7, 42.9%) not differentiating between procedural/medication and control treatment groups. Three studies (75%) reported greater adverse events in combination vs. monotherapy groups. Information on cost was not provided in any study.

## 4. Discussion/Conclusions

Our study showed that medication-only combination therapy showed efficacy compared to monotherapy for onychomycosis treatment in more than half of trials, with limited adverse events. However, seven studies had conflicting results and in studies that showed greater efficacy for monotherapy vs. combination therapy, significance was not sustained at later endpoints [26] or was only observed in the groups with longer treatment durations (i.e., oral terbinafine 12 vs. 6 weeks) [20,21]. Importantly, trial lengths were relatively short, with average follow-up of 46.1 weeks (SD: 21.5; range: 24–78.2 weeks). In contrast, pivotal RCTs on oral terbinafine [8,48,49,50,51,52,53,54,55,56,57,58,59,60,61,62,63], oral itraconazole [8,50,52,53,54,56,58,59,60,63,64,65,66,67], and topical efinaconazole [68,69,70,71] had average follow-ups of 69.0 (SD: 49.1; range: 36–252), 66.4 (SD: 55.8; range: 19–252), and 62.1 (SD: 28.7; range: 40–104.3) weeks, respectively. Taken together, medicine-only combination therapy trials were much less rigorously designed than pivotal monotherapy trials, and therefore should be interpreted with caution.

We found that seven medication-based studies yielded conflicting results, with similar quality across studies. Two studies showed conflicting results for oral itraconazole and topical amorolfine therapy, with one study including 131 patients, multicenter, and 5.5 months of follow-up [28], versus 90 patients, single-center, and 9 months of follow-up [29]. Three studies showed conflicting results for oral terbinafine and topical ciclopirox combination therapy, with one study including 80 patients, single-center, 9 months of follow-up, and non-blinded [23], another including 73 patients, multi-center, 11 months of follow-up, and single-blinded [24], and a final study including 96 patients, single-center, 8.3 months of follow-up, and single-blinded [25]. Two studies had conflicting results on oral itraconazole and oral terbinafine combination therapy, with one study including 190 patients, multicenter, 16.6 months of follow-up, and single-blinded [30], versus 106 patients, multicenter, and single-blinded [31]. Given that these trials with conflicting results were comparable in terms of quality, without a clearly superior trial demonstrating greater efficacy for combination vs. monotherapy treatments, large, multicenter, double-blinded trials with sufficient follow-up are necessary to determine the efficacy of combination therapy for onychomycosis treatment.

In three medication-only studies that provided cost information, combination therapy was more cost effective than monotherapy, considering duration of usage and efficacy. However, this data cannot be extrapolated to include combination oral therapy with the newer topicals. In a review of data from the National Average Drug Acquisition Cost Medicaid Pharmacy Pricing database, 2013–2018 [72], inflation-adjusted costs changed at an annual rate of −18.2% and −3.4% for generic oral itraconazole and terbinafine, respectively, while brand name medications Kerydin, Jublia, and Diflucan increased 3.7%, 4.5%, and 17.2%, respectively. This data suggests that branded topical and oral antifungals are costly, and that prescribing them as part of combination regimens will increase health care costs. In a study evaluating the cost of topical efinaconazole 10% solution [73], a 48-week treatment course for one great toenail ($8057) was nearly 35 and 12 times more expensive than projected costs (using data from goodrx.com, accessed on 1 July 2021) of 3 months of treatment with oral terbinafine (250 mg/day, $233) and oral itraconazole (200 mg/day, $683), respectively, without consideration of relative efficacy. In an analysis of Medicare provider utilization and payment data, part D, 2013–2018 [74], total costs and costs per supply day increased yearly by 3091% and 144%, respectively, for topical efinaconazole, and decreased and increased by 12.5% and 42.4%, respectively, for topical tavaborole. Therefore, more research is necessary to assess cost vs. benefits of combination treatments compared to monotherapy.

We found that almost all studies examining combination procedural and medication therapy showed significant benefit compared to monotherapy. However, trials were relatively short, with an average follow-up of 32.5 weeks (SD: 14.3; range: 13.04–62.0 weeks). RCTs on photodynamic and laser monotherapies had a similar average follow-up of 35.3 weeks (SD: 11.5; range: 24.0–52.0 weeks) [75,76,77,78,79,80,81,82,83,84,85], which suggests that procedural studies, in general, lack the long-term follow-up that is sufficient to determine efficacy of onychomycosis treatment. Furthermore, in our review, some monotherapy and combination arms did not demonstrate efficacy for severe onychomycosis cases [35]. In a review of 24 laser trials on onychomycosis [86], there was limited evidence supporting lasers for onychomycosis cure, and only 30% and 20% of RCTs described methods of randomization or utilized blinding in their experimental design, respectively. Furthermore, in a systematic review of 25 RCTs investigating laser monotherapy for toenail onychomycosis [87], mycological cure was evaluated in only one study, and complete cure was not reported in any study. Mean OSI changes from baseline were minimal (range: −3.6. to +1.4), and efficacies of control and treatment groups were similar, thereby failing to demonstrate improvements in the US FDA approved endpoint “temporary increase of clear nail” [3]. Methodology of current laser trials is inadequate, both for monotherapy trials and for combination trials reported in this review. Therefore, laser monotherapy efficacy should be confirmed in rigorous RCTs, before it can be considered for use in conjunction with other onychomycosis medications.

While cost was not reported in any procedural and medication combination trials evaluated in this review, laser treatments are expensive, not covered by insurance, and typically require multiple monthly sessions [3]. Considering that laser therapies are less efficacious than topical or oral therapeutic options [6] and are costly, we caution against use of lasers in combination with antifungals until more robust RCTs are conducted, demonstrating superior efficacy with favorable cost–benefit profiles.

We found that combination therapy was generally well tolerated across medication-only studies. Notably, in a systematic review and meta-analysis of 26 RCTs investigating monotherapy for toenail onychomycosis [5], the OR of adverse events in any treatment group did not significantly differ from placebo, except in the case of efinaconazole 10% solution (OR 1.28; 95% CI: 1.02–1.61), for transient application-site reactions. Therefore, since monotherapy for onychomycosis treatment is well tolerated, we recommend single medication therapy to limit adverse effects. In procedural and medication studies, we found greater reports of adverse events with combination therapy than monotherapy, however, sample sizes were small, and in all three studies, monotherapy was a topical or placebo treatment, rather than a procedural treatment. It is therefore uncertain whether the adverse event in the combination group was due to the procedure itself (i.e., skin irritation from laser treatment), or due to the combination of treatments. In a systematic review of 35 RCTs (1723 patients and 4278 nails with onychomycosis) [88], the majority of patients reported a mild-to-moderate burning sensation during laser treatment, with some reporting bleeding.

In pivotal RCTs investigating oral terbinafine monotherapy, adverse effects were transient, mild to moderate in severity, and not significantly different from placebo groups [89]. There were no reported laboratory abnormalities [90]. In the terbinafine package insert, liver enzyme abnormalities and taste disturbances were only reported in 3.3% and 2.8% of patients, respectively, with a discontinuation rate of 0.2% for both (https://www.accessdata.fda.gov/drugsatfda_docs/label/2012/020539s021lbl.pdf, accessed on 18 January 2022). Oral itraconazole monotherapy is well tolerated, with the most common reported adverse events being gastrointestinal discomfort and headache, both mild and transient [66], and with safety profiles similar between placebo and itraconazole treatment groups in a phase-III RCT [64]. In two phase-III multicenter RCTs assessing topical efinaconazole monotherapy [68], rates of adverse events were similar between treatment and vehicle groups in both studies, with most mild or moderate in severity, low rates of treatment-related discontinuation, and no clinically meaningful laboratory or vital sign changes from baseline. Therefore, when used as monotherapy, FDA-approved onychomycosis treatments have limited side effects, with none that are life threatening, and rarely lead to medication discontinuation. However, it is important to note that oral itraconazole and fluconazole are contraindicated with certain medications. In addition, oral itraconazole, fluconazole, and terbinafine, can alter the plasma concentration of select medications [6]. Considering the frequency of drug interactions of oral onychomycosis therapies with other medications, initiation of oral combination therapy would therefore require close monitoring of patients on multiple medications, with medication adjustments or dose reductions more likely than for patients on monotherapy treatment.

In a meta-analysis of 26 RCTs investigating systemic monotherapy for toenail onychomycosis [5], there was significantly greater OR of achieving mycological cure for all treatments vs. placebo, with continuous itraconazole 200 mg (OR: 18.61; 95% CI: 7.40–46.81) and continuous terbinafine 250 mg (OR: 16.41; 95% CI: 6.49–41.47) the most efficacious treatments. Monotherapy therefore is effective in treating onychomycosis. Therefore, taken together with our analysis of combination therapy for onychomycosis, monotherapy should be considered as a first-line treatment option prior to initiating combination therapy.

Poor prognostic factors for onychomycosis treatment include patient characteristics (older age, history of personal history of onychomycosis), comorbidities (immunosuppression, peripheral vascular disease, uncontrolled diabetes mellitus), nail characteristics (prior nail trauma, proximal subungual onychomycosis, dermatophytoma, severe onycholysis), and infecting organism (mixed fungal infection, yeasts, non-dermatophytes) [6].

Predisposition to mucocutaneous fungal infections may be due to specific genotypes. For example, Tyr238X dectin-1 or caspase recruitment domain containing protein-9 mutations cause impaired β-glucan recognition and cytokine responses, and defects in major histocompatibility complexes may interfere with initial fungi recognition and prevent T-cell activation. Alterations in intercellular adhesion molecule-1 may prevent immune cells from migrating to infected tissues, and elevated levels of T-regulatory cells may modify T-cell behavior, all of which may increase susceptibility to dermatophyte and *Candida* spp. infections [91,92].

In patients with poor prognostic factors, the advantages of combination therapy may outweigh the risks and costs, and should be considered especially in patients who failed previous treatments. Importantly, we found that in many combination trials, patients with poor prognostic factors or risk factors for recurrence were excluded. In medication-only studies, six provided no information on exclusion criteria, while 66.7% (6/9) of studies with exclusion criteria included a risk factor for poor prognosis or high risk of recurrence. There was an upper age limit for inclusion in the study in 20% (3/15) of studies. In procedural and medication studies, all studies included information on exclusion criteria, with 66.7% (10/15) including a risk factor for poor prognosis or high risk of recurrence. Upper age limits for inclusion were reported in 26.7% (4/15) of studies. Medication-based monotherapy trials showed good efficacy in some subgroups, including older adults [52], diabetics [75,93,94,95], and those with severe onychomycosis [66] or dermatophytoma [96]. Therefore, published combination trials excluded many patients who may have benefited most from combination therapy, such as older patients and the immunocompromised, and future trials must be conducted in these patient populations to determine the efficacy of combination therapy in difficult-to-treat onychomycosis cases.

Considering the limited number of studies available for review, conflicting results in medication-only based studies, limited efficacy of procedural combination treatments, and cost considerations, we recommend that combination therapy be considered as a second-line treatment option for patients with resistant cases of onychomycosis or poor prognostic features. Our data showed that combinations of oral griseofulvin and topical tioconazole or bifonazole, or oral terbinafine and topical amorolfine, demonstrated significant improvements in combination vs. monotherapy group in all trials. Oral terbinafine, itraconazole, and griseofulvin are US FDA approved for onychomycosis treatment, however, griseofulvin is no longer commonly used, considering its inferior efficacy compared to other treatment options, lengthier treatment courses, and higher risk of adverse effects [3]. Topical efinaconazole 10% and tavaborole 5% solution are US FDA approved for toenail onychomycosis, with ciclopirox 8% nail lacquer approved for both fingernail and toenail onychomycosis [6], and topical tioconazole, bifonazole, and amorolfine are not currently available in the US. A panel of expert dermatologists, podiatrists, and a microbiologist [4], recommended that terbinafine and efinaconazole 10% solution should be used as first-line oral and topical onychomycosis treatments, respectively. Considering these recommendations, the results from our study, and accessibility of antifungal treatments in the US, we recommend oral terbinafine as first line, used alone for straightforward moderate to severe onychomycosis cases, and in combination with efinaconazole 10% solution, tavaborole 5% solution, or ciclopirox 8% nail lacquer for patients who failed previous treatments or who have poor prognostic factors. Nonetheless, medication selection must be tailored to each individual patient with consideration of the extent of nail involvement, infecting pathogen, comorbidities, concomitant medications, expense, and patient preferences [6].

Retinoids in combination with antifungals may be effective for onychomycosis treatment. Retinoids have both in vitro and in vivo antimicrobial activity against fungi, with tretinoin and isotretinoin most effective against *M. furfur*, tazarotene against dermatophytes, and tretinoin against *A. fumigatus* and *C. albicans*. By preventing hyphal germination necessary for biofilm formation, all-trans retinoic acid may also be effective against *Candida* biofilm-related infections, which are typically difficult to treat due to multidrug resistance [97]. In a study performed after our systematic search on 135 patients with toenail and/or fingernail onychomycosis receiving oral itraconazole pulse monotherapy, oral acitretin monotherapy, or combined pulsed itraconazole and acitretin for 3 months [98], mycological cure was 51.1%, 28.9%, and 80%, respectively, and complete cure was 20%, 28.9%, and 53.3%, respectively (*p* ≤ 0.05). OSI scores significantly improved in the combination group compared to itraconazole monotherapy (*p* = 0.005) and acitretin monotherapy (*p* = 0.006) groups, with no difference observed between the monotherapy groups (*p* = 0.95). Therefore, retinoids combined with antifungals may increase efficacy compared to monotherapy treatments. Large RCTs are needed to corroborate these findings.

There are several limitations to our study. While we performed an exhaustive search of combination studies for onychomycosis treatment, only a small number met inclusion criteria for our review. Different endpoints and medication combinations were used across studies, limiting the ability to make direct comparisons of study findings. It is also difficult to draw comparisons about studies that use keratolytic agents in combination with topical antifungals. Furthermore, study inclusion/exclusion criteria differed across studies (Table 3). Nail characteristics variably specified fingernail vs. toenail, number of affected nails, matrix involvement, and nail plate surface area and thickness. *T. rubrum* was the etiological agent in most studies, while others did not list the fungal organisms, or reported mixed infections. Vehicles differed, including nail lacquers, creams, and solutions, likely contributing to differences in efficacy. The majority of studies were conducted in Europe or North America, limiting the diversity of participants.

Future research should be directed at conducting combination clinical trials that are large, multicenter, and randomized, with sufficient follow-up, and including diverse patient populations across different geographic locations, to determine the value of combination therapy for treatment of onychomycosis, and to establish standard treatment regimens. Until such studies are conducted, and a clear clinical benefit of combination therapy is demonstrated, we recommend combination therapy as a second-line option in patients with difficult-to-treat cases or poor prognostic factors.

## Figures and Tables

**Table 1:** Treatment protocols and outcomes for studies investigating medication-only based combination therapies for onychomycosis treatment.

Study	Combination Therapy	# Subjects Completed	Combination Treatment Protocol	Monotherapy Treatment Protocol	Combination Treatment Rates, %	Monotherapy Treatment Rates, %	Difference between Groups	Adverse Effects Combination Therapy, *n*, %	Adverse Effects Monotherapy, *n*, %
Hay, R. J. et al., 1987 [18] †	Oral griseofulvin & topical tioconazole	10	Oral griseofulvin 1 g daily + topical tioconazole 28% on 1 affected side × 12 months	Oral griseofulvin 1 g daily + placebo on other affected side × 12 months	12 months CO: 69	12 months CO: 41	*p* < 0.005 *	N/a	1, 20
Friedman-Birnbaum, R. et al., 1997 [19] †	Oral griseofulvin & topical bifonazole	98	Oral griseofulvin 500 mg daily + topical bifonazole 1% cream × 4 weeks	Oral griseofulvin 500 mg daily + placebo × 4 weeks	4 months M: 93 RL: 7 CO: 43.7	4 months M: 66 RL: 20 CO: 20	M: *p* < 0.01 * RL: *p* < 0.01 *	5, 8.5	1, 1.7
Baran, R. et al., 2000 [20] †	Oral terbinafine & topical amorolfine	145	Topical amorolfine 5% once weekly × 15 months plus oral terbinafine 250 mg daily × 6 weeks (AT6) or 12 weeks (AT12)	Oral terbinafine 250 mg daily × 12 weeks (T12)	3 months M: 35 (AT6), 27.5 (AT12) 18 months CO: 44 (AT6), 72.3 (AT12)	3 months M: 17.1 18 months CO: 37.5	CO: 95% CI 57.4–84.4 (AT12) vs. 23.9–52.6 (T12) *	AT6: 21, 42 AT12: 23, 49	23, 48
Baran, R. 2001 [21] †	Oral terbinafine & topical amorolfine	145	Topical amorolfine 5% once weekly × 15 months plus oral terbinafine 250 mg daily × 6 weeks (AT6) or 12 weeks (AT12)	Oral terbinafine 250 mg daily × 12 weeks (T12)	3 months M: 35 (AT6), 27.5 (AT12) 18 months C: 46 (AT6), 74 (AT12) CO: 44 (AT6), 72.3 (AT12)	3 months M: 17.1 18 months C: 42 CO: 37.5	C: 95% CI 60–86 (AT12), 28–57 (T12) * CO: 95% CI 57–84 (AT12), 24–53 (T12) *	Not specified by group (*n* = 34 total)	Not specified by group (*n* = 34 total)
Baran, R. et al., 2007 [22] †	Oral terbinafine & topical amorolfine	208	Oral terbinafine 250 mg daily × 3 months + amorolfine hydrocholoride 5% nail lacquer once weekly × 12 months	Oral terbinafine 250 mg daily × 3 months	3 months M: 94.2 18 months C: 66.7 CO: 59.2	3 months M: 59.7 18 months C: 53.5 CO: 45	M: *p* < 0.001 * C: *p* < 0.04 * CO: *p* = 0.03 *	19, 15.9	15, 11.6
Avner, S. et al., 2005 [23] †	Oral terbinafine & topical ciclopirox	68	Oral terbinafine 250 mg daily × 16 weeks + topical ciclopirox nail lacquer daily × 9 months	Oral terbinafine 250 mg daily × 16 weeks	9 months M: 88.2 CO: 67.7 CS: 82.4	9 months M: 64.7 CO: 50 CS: 58.8	M: *p* = 0.043 * CO: *p* = 0.218 N: *p* = 0.004 *	N/a	N/a
Gupta, A. K. et al., 2005 [24]	Oral terbinafine & topical ciclopirox	63	Pulse: oral terbinafine 250 mg × 4 weeks, 4-week rest, 4-weeks on + ciclopirox nail lacquer daily × 48 weeks Continuous: oral terbinafine 250 mg daily × 12 weeks + ciclopirox nail lacquer daily × 48 weeks	Oral terbinafine 250 mg daily × 12 weeks	48 weeks M: 66.7 (pulse), 70.4 (continuous) E: 40.0 (pulse), 33.3 (continuous)	48 weeks M: 56.0 E: 34.8	M: *p* > 0.05 E: *p* > 0.05	4.3, 20.5 (pulse), 5.8, 21.4 (continuous)	5.5, 22.0
Jaiswal, A. et al., 2007 [25]	Oral terbinafine & topical ciclopirox or amorolfine	92	(A) Oral terbinafine pulse therapy + topical ciclopirox olamine 8% once daily × 4 months (B) Oral terbinafine pulse therapy + topical amorolfine hydrocholoride 5% once weekly × 4 months	Oral terbinafine 250 mg twice daily × 7 days for 4 months (pulse therapy)	9 months C: 82.6 (A), 73.91 (B) M: 83.3 (A), 70 (B)	9 months C: 71.73 M: 82.6	C: *p* > 0.05 M: *p* > 0.05	Not specified by group (*n* = 14 total)	Not specified by group (*n* = 14 total)
Tietz, H. J. et al., 2013 [26] †	Topical bifonazole & urea	595	Nail detachment with urea 40% paste applied daily for 14–28 days + topical bifonazole 1% applied daily × 28 days	Nail detachment with urea 40% paste applied daily for 14–28 days + topical placebo cream applied daily × 28 days	2 weeks CO: 54.8 M: 64.5 C: 86.6 3 months CO: 50.7 M: 61.5 C: 73.8 6 months CO: 33.6 M: 52.1 C: 56.8	2 weeks CO: 42.2 M: 49.0 C: 82.8 3 months CO: 40.9 M: 49.1 C: 73.7 6 months: CO: 34.6 M: 48.1 C: 56.8	2 weeks CO: *p* = 0.0024 * M: *p* = 0.0001 * C: *p* = 0.2109 3 months CO: *p* = 0.0260 * M: *p* = 0.0033 * C: *p* = 1.0 6 months CO: *p* = 0.8581 M: *p* = 0.3568 C: *p* = 1.0	Treatment phase: 12, 3.7 Follow-up: 35, 10.8	Treatment phase: 18, 5.5 Follow-up: 43, 13.1
Bassiri-Jahromi et al., 2012 [27]	Topical fluconazole & urea	66	Topical fluconazole 1% + urea 40% once daily × 6 months	Topical fluconazole 1% once daily × 6 months	6 months M: 82.8 CR: 77.1	6 months M: 62.6 CR: 68	Not reported	Not specified by group (*n* = 1 total)	Not specified by group (*n* = 1 total)
Lecha, M. 2001 [28] †	Oral itraconazole & topical amorolfine	114	Amorolfine 5% nail lacquer once weekly × 24 weeks + oral itraconazole 200 mg daily × 6 weeks (A) or 12 weeks (B)	Oral itraconazole 250 mg daily × 12 weeks	12 weeks M: 93.3 (A), 82.9 (B) 24 weeks M: ≥90 (A & B) C: 88.1 (A), 100 (B) CO: 83.7 (A), 93.9 (B)	12 weeks M: 41.2 24 weeks M: <69 C: 90.3 CO: 68.8	12 weeks M: *p* < 0.001 * (A & B vs. monotherapy) 24 weeks M: *p* < 0.001 * CO: *p* = 0.011 * (A & B vs. monotherapy)	Not specified by group (*n* = 21 total)	Not specified by group (*n* = 21 total)
Rigopoulos, D. et al., 2003 [29]	Oral itraconazole & topical amorolfine	85	Oral itraconazole 400 mg daily × 1 week at 3-week intervals (pulse therapy) × 2 months + topical amorolfine 5% solution nail lacquer once weekly × 6 months	Oral itraconazole × 3 pulses	3 months M: 74 9 months CO: 93	3 months M: 60 9 months CO: 91	M: *p* > 0.1 CO: *p* > 0.1	N/a	N/a
Gupta, A. K. et al., 2001 [30] †	Oral itraconazole & terbinafine	165	Two pulses oral itraconazole (200 mg twice daily × 1 week) + 1 or 2 pulses oral terbinafine (250 mg twice daily × 1 week)	Three or four pulses oral terbinafine	72 weeks M: 66.7 C: 51.9 CO: 48.1 E: 60.5	72 weeks M: 46.3 C: 36.8 CO: 30.5 E: 43.2	M: *p* = 0.007 * C: *p* = 0.09 CO: *p* = 0.03 * E: *p* = 0.02 *	12, 16.0	22, 24.4
Gupta, A. K. et al., 2013 [31]	Oral itraconazole & terbinafine	149	Oral itraconazole 200 mg daily for weeks 1–4 & oral terbinafine 250 mg daily for weeks 3–6	(A) Oral terbinafine 250 mg daily × 12 weeks (B) Oral terbinafine 250 mg/day 4 weeks on, 4 weeks off, 4 weeks on (C) Oral itraconazole 200 mg twice daily pulse therapy (7 days on, 21 days off) × 3 pulses	After 48 weeks MR: 57 R: 67	After 48 weeks MR: (A) 32 (B) 36 (C) 59 R: (A) 40 (B) 50 (C) 50	MR: *p* = 0.085 R: *p* = 0.711	N/a	N/a
Arenas, et al., 1991 [32]	Oral itraconazole or griseofulvin & topical isoconazole or urea	83	Oral griseofulvin 500 mg daily + topical isoconazole 1% twice daily (A) or urea 40% occlusive patch (B) × 6 months Oral itraconazole 100 mg daily + topical isoconazole 1% twice daily (C) or urea 40% occlusive patch (D) × 6 months	Topical placebo cream + oral griseofulvin 500 mg daily (E) or oral itraconazole 100 mg daily (F) × 6 months	6 months M: (A) 46.1 (B) 42.8 (C) 73.3 (D) 78.5 Overall griseofulvin (A, B, E): 38.09 Overall itraconazole (C, D, F): 80.48	6 months M: (E) 26.6 (F) 91.65	6 months M: *p* = 0.010 Overall M (griseofulvin vs. itraconazole): *p* = 0.001 *	N/a	N/a

C: clinical cure rate; CI: confidence interval; CO: complete cure rate; CR: clinical improvement rate; CS: clinical status, marked improvement or cured; E: effective therapy rate; M: mycological cure rate; MR: mycological recurrence rate; N/a: not applicable; R: recurrence rate; RL: relapse rate. * Significant difference between treatment groups. † Studies showing significant benefit of combination therapy over monotherapy.

**Table 2:** Treatment protocols and outcomes for studies investigating procedures (debridement, photodynamic therapy, or lasers) in combination with medication for onychomycosis treatment.

Study	Combination Therapy	# Subjects Completed	Combination Treatment Protocol	Monotherapy Treatment Protocol	Combination Treatment Rates, %	Monotherapy Treatment Rates, %	Difference between Groups	Adverse Effects Combination Therapy, *n*, %	Adverse Effects Monotherapy, *n*, %
Zhang, J. et al., 2016 [33] †	Laser & topical amorolfine	9	2940-nm fractional Er:YAG laser once weekly at weeks 1, 2, 3, 4, 8, & 12 + 5% amorolfine lacquer twice weekly × 12 weeks	Amorolfine 5% lacquer twice weekly × 12 weeks	12 weeks M: 70 24 weeks M: 75	12 weeks M: 25 24 weeks M: 20	12 weeks: *p* = 0.01 * 24 weeks: *p* = 0.001 *	Not specified by group (*n* = 3 total)	Not specified by group (*n* = 3 total)
Bunyaratevej, S. et al., 2020 [34] †	Laser & topical amorolfine	60	(A) Long-pulsed Nd:YAG 1064-nm laser × 4 sessions at 1 month intervals + topical amorolfine nail lacquer × 3 months	(B) Nd:YAG 1064-nm laser × 4 sessions at 1 month intervals (C) Topical amorolfine nail lacquer × 3 months	3 months M: 65 C: 30	3 months M: 35 (B), 60 (C) C: 10 (B), 30 (C)	M: *p* = 0.05 * (A vs. B)	N/a	N/a
Zhang, J. et al., 2021 [35] †	Laser & topical amorolfine	78	2940-nm Er:YAG fractional laser × 6 treatments at weeks 1, 2, 3, 4, 8, & 12 + topical amorolfine 5% nail lacquer twice weekly × 12 weeks	Topical amorolfine 5% lacquer twice weekly × 12 weeks	Mild (A), moderate (B), severe (C) onychomycosis 12 weeks M: 100 (A), 63.64 (B), 7.69 (C) O: 2 (A), 6, (B) 4 (C) 24 weeks M: 100 (A), 66.67 (B), 7.79(C) O: 2 (A), 8 (B), 4 (C)	Mild (A), moderate (B), severe (C) onychomycosis 12 weeks M: 84.62 (A), 38.24 (B), 8.33 (C) O: 1 (A), 3 (B), 7 (C) 24 weeks M: 61.54 (A), 35.29 (B), 4.17 (C) O: 1 (A), 4 (B), 8 (C)	12 weeks M: *p* = 0.038 * (B) O: *p* = 0.037 * (A), *p* < 0.001 * (B) 24 weeks M: *p* = 0.046 * (A), *p* = 0.01 * (B) O: *p* = 0.002 * (A), *p* < 0.001 * (B)	32, 84.213	N/a
Bonhert, K. et al., 2019 [36] †	Laser & topical efinaconazole	30	1064-nm Nd-YAG laser × 6 treatments spaced 4 weeks apart + topical efinaconazole 10% once daily × 48 weeks	Topical efinaconazole 10% once daily × 48 weeks	48 weeks M: 90 52 weeks M: 92	48 weeks M: 70 52 weeks M: 86	Combined vs. monotherapy: - Quicker overall improvement at weeks 24 (*p* = 0.04 *), 36, 48 (both *p* = 0.03 *), and 52 (*p* = 0.02 *) - Greater improvement in SCIO index at weeks 36, 48 (both *p* = 0.04 *), & 52 (*p* = 0.02)	7, 46%	N/a
Li, Y. et al., 2016 [37] †	Laser & oral itraconazole	19	1064-nm Nd:YAG laser once weekly × 8 weeks + 200 mg oral itraconazole twice daily × 1 week for 4 times	1064-nm Nd:YAG laser once weekly × 8 weeks for 4 times	Mild/moderate (A), severe onychomycosis (B) 8 weeks E: 21 (A), 20 (B) 16 weeks E: 20 (A), 19 (B) 24 weeks E: 19 (A), 21 (B)	Mild/moderate (A), severe onychomycosis (B) 8 weeks E: 17 (A), 13 (B) 16 weeks E: 17 (A), 14 (B) 24 weeks E: 19 (A), 11(B)	A: *p* > 0.05 (8, 16, 24 weeks) B: *p* < 0.05 * (8 & 24 weeks), *p* > 0.05 (16 weeks)	N/a	N/a
Hamed Khater, M. & Khattab, F.M. 2020 [38] †	Laser & oral itraconazole	30	1064-nm long-pulsed Nd-YAG laser × 6 sessions (every 2 weeks × 3 months) + oral itraconazole 200 mg twice daily 1 week per month × 3 months	Oral itraconazole 200 mg twice daily 1 week per month × 3 months	C: Excellent: 66.6 Moderate: 6.6 Good: 20.1 Mild: 6.6 MR: Excellent: 13.3 Moderate: 40.6 Good: 13.3 Mild: 33.3 Mean OSI after treatment: 5.07 ± 4.15	C: Excellent: 13.3 Moderate: 33.3 Good: 40.1 Mild: 13.3 MR: Excellent: 13.3 Moderate: 40.1 Good: 13.3 Mild: 33.3 Mean OSI after treatment: 6.67± 3.60	Overall C: *p* = 0.001 * Mean OSI: *p* < 0.01 *	N/a	N/a
Zaki, A.M. et al., 2020 [39] †	Laser & topical tioconazole	120	Fractional CO_2_ laser × 5 sessions at 3-weeks intervals + topical tioconazole 28% applied twice daily × 16 weeks	(A) Fractional CO_2_ laser × 5 sessions at 3-weeks intervals × 16 weeks (B) Topical tioconazole 28% applied twice daily × 16 weeks	C: 55 PS: 60 KOH turned negative: 80 Culture turned negative: 70	C: 30 (A), 25 (B) PS: 40 (A), 30 (B) KOH turned negative: 60 (A), 55 (B) Culture turned negative: 50 (B), 30 (C)	C: *p* < 0.001 * PS: *p* = 0.007 * KOH turned negative: *p* = 0.001 * Culture turned negative: *p* < 0.001 *	N/a	N/a
Zhou, B.R. et al., 2016 [40] †	Laser & topical luliconazole	60	Fractional CO_2_ laser × 12 sessions at 2-weeks intervals + luliconazole 1% cream daily × 6 months	Fractional CO_2_ laser × 12 sessions at 2-week intervals × 6 months	3 months C: 69.6 6 months C: 73.0 3 months after last treatment M: 69.6	3 months C: 50.9 6 months C: 52.8 3 months after last treatment M: 57.4	C: *p* = 0.004 * (3 months), *p* = 0.002 * (6 months) M: *p* = 0.006 *	N/a	N/a
Xu, Y. et al., 2014 [41] †	Laser & oral terbinafine	53	Long-pulsed 1064-nm Nd:YAG laser treatment once weekly + oral terbinafine 250 mg daily × 24 weeks	(A) Long-pulsed 1064-nm Nd:YAG laser treatment once weekly × 24 weeks (B) Oral terbinafine 250 mg daily × 24 weeks	4 weeks M: 31.03 C: 20.69 8 weeks M: 68.97 C: 51.72 12 weeks M: 93.10 C: 86.21 16 weeks M: 96.55 C: 93.10 24 weeks M: 100 C: 96.55	4 weeks M: 0 (A), 10 (B) C: 0 (A), 0 (B) 8 weeks M: 16.13 (A), 36.67 (B) C: 3.23 (A), 16.67 (B) 12 weeks M: 35.48 (A), 70 (B) C: 29.03 (A), 63.33 (B) 16 weeks M: 48.39 (A), 73.33 (B) C: 35.48 (A), 70 (B) 24 weeks M: 77.42 (A), 83.33 (B) C: 64.52 (A), 73.33 (B)	M, C (combination vs. A & B): *p* < 0.05 * (all timepoints)	N/a	1, 6.3 (B)
Kim, T.I. et al., 2016 [42] †	Laser & topical naftifine HCl spray	53	1064-nm Nd:YAG laser × 3 sessions at 4-week intervals + topical naftifine HCl spray daily × 24 weeks	(A) 1064-nm Nd:YAG laser × 3 sessions at 4-week intervals (B) Naftifine HCl spray daily × 24 weeks	12 weeks C: 35.2 M: 14.1 24 weeks C: 40.8 M: 22.5	12 weeks C: 25.3 (A), 7.5 (B) M: 8.9 (A), 6.0 (B) 24 weeks C: 31.6 (A), 7.5 (B) M: 15.2 (A), 4.5 (B)	C: *p* < 0.005 * (combination/A vs. B at 12 and 24 weeks) M: *p* < 0.005 * (combination/A vs. B at 24 weeks)	N/a	N/a
Gilaberte, Y. et al., 2017 [43]	Methyl aminolevulinate photodynamic therapy & topical urea	40	Methyl aminolevulinate photodynamic therapy + urea 40% ointment × 3 sessions	Placebo (red light) photodynamic therapy + urea 40% ointment × 3 sessions	M: 31.82 CO: 18.18	M: 11.1 CO: 31.82	M: *p* = 0.178 CO: *p* = 0.23	Pigmentation: 22, 100 Inflammation: 4, 18.2 Tinea pedis: 3, 13.64	Pigmentation: 15, 83.3 Inflammation: 0, 0 Tinea pedis: 2, 11.11
Jennings, M.B. et al., 2006 [44] †	Debridement & oral terbinafine	504	Oral terbinafine 250 mg daily × 12 weeks + aggressive nail debridement	Oral terbinafine 250 mg daily × 12 weeks	48 weeks C: 59.8 M: 67.5 CO: 37.8	48 weeks C: 51.4 M: 62.6 CO: 32.5	C: *p* = 0.023 * M: *p* > 0.05 CO: *p* > 0.05	Not specified by group (*n* = 116 total)	Not specified by group (*n* = 116 total)
Potter, L.P. et al., 2007 [45] †	Debridement & oral terbinafine	504	Oral terbinafine 250 mg daily × 12 weeks + aggressive nail debridement at baseline & weeks 6, 12, & 24	Oral terbinafine 250 mg daily × 12 weeks	SF: 28.7 SB: 20.4 A: 25.5 PA: 20.7 OP: 28.2 S: 8.9	SF: 25.8 SB: 19.2 A: 23.4 PA: 20.7 OP: 28.2 S: 10.0	SF: *p* = 0.0395 * SB: *p* = 0.3783 A: *p* = 0.1543 PA: *p* = 0.9761 OP: *p* = 0.9897 S: *p* = 0.4040 TS: *p* = 0.0077 *	N/a	N/a
Malay, D.S. et al., 2009 [46] †	Debridement & topical ciclopirox	55	Debridement at 3-month intervals × 9–12 months + topical ciclopirox 8% daily	Debridement at 3-month intervals × 9–12 months	M: 76.74	M: 0	M: *p* < 0.05 *	N/a	N/a
Shemer, A. et al., 2016 [47] †	Nail drilling, oral & topical terbinafine	98	(A) Nail drilling once at baseline + oral terbinafine 250 mg daily × 2 weeks + topical terbinafine 1% spray twice daily × 6 month (B) Nail drilling once at baseline + topical terbinafine 1% spray twice daily × 6 months	Topical terbinafine 1% spray twice daily × 6 months	10 weeks M: 14.3 (A), 2.4 (B) 16 weeks M: 35.7 (A), 6.3 (B) CN: 63.75 (A), 39.95 (B) 22 weeks M: 46.2 (A), 32.4 (B) CN: 59.38 (A), 52.39 (B) 28 weeks M: 47.1 (A), 34.2 (B)	10 weeks M: 0.0 16 weeks M: 0.0 CN: 31.36 22 weeks M: 5.0 CN: 23.81 28 weeks M: 8.0	16 weeks CN: *p* = 0.028 * (A vs. C) 22 weeks CN: *p* = 0.005 * (A vs. C), *p* = 0.014 * (B vs. C)	Not specified by group (*n* = 8 total)	Not specified by group (*n* = 8 total)

A: appearance problems; C: clinical cure rate; CI: confidence interval; CN: mean percent clear nail; CO: complete cure rate; CR: clinical response rate; D: mean diameter of inhibition zone (mm); E: efficacy rate; HCl: hydrochloride; KOH: potassium hydroxide; M: mycological cure rate; MR: mycological recurrence rate; N: no clinical improvement rate; S: stigma; N/a: not applicable; NC: negative culture; O: decrease in onychomycosis severity index score; OP: overall problem; OSI: onychomycosis severity index score; PA: physical activities problems; PS: patient satisfaction; R: recurrence rate; RL: relapse rate; S: stigma; SB: symptom bothersomeness; SCIO: Scoring Clinical Index for Onychomycosis; SF: symptom frequency; TS: treatment satisfaction. * Significant difference between treatment groups. † Studies showing significant benefit of combination therapy over monotherapy.

**Table 3:** Nail characteristics, pathogens, topical vehicles, and study location for all 30 studies.

Study	Combination Therapy	Pathogen(s)	Fingernail or Toenail Involvement	Number of Affected Nail(s) for Inclusion	Nail Characteristics for Inclusion	Topical Vehicles	Study Location
Hay, R. J. et al., 1987 [18]	Oral griseofulvin & topical tioconazole	*T. rubrum* (100%)	Bilateral toenails	N/A	N/A	Nail solution	United Kingdom
Friedman-Birnbaum, R. et al., 1997 [19]	Oral griseofulvin & topical bifonazole	*T. rubrum* (93%), *T. tonsurans* (4%), *T. mentagrophytes* (3%)	93% toenail, 7% fingernail	N/A	N/A	Cream	Israel
Baran, R. et al., 2000 [20]	Oral terbinafine & topical amorolfine	*T. rubrum* (98%), *T. interdigitale* (1.4%), *T. soudanense* (0.7%)	Toenails	N/A	Matrix involvement	Nail lacquer	France
Baran, R. 2001 [21]	Oral terbinafine & topical amorolfine	*T. rubrum* (98%), *T. interdigitale* (1.4%), *T. soudanense* (0.7%)	Toenails	At least 1 (not including little toenail)	≥80% of the nail plate surface area and/or matrix involvement	Nail lacquer	France
Baran, R. et al., 2007 [22]	Oral terbinafine & topical amorolfine	*T. rubrum* (93.3%), *T. mentagrophytes* (4.4%), *S. brevicaulis* (0.8%), *T. interdigitale* & *Acremonium* spp. (both 0.4%)	Toenails	At least 1 great toenail	Matrix involvement	Nail lacquer	Europe
Avner, S. et al., 2005 [23]	Oral terbinafine & topical ciclopirox	*T. rubrum* (95.6%), *T. mentagrophytes* (4.4%)	Toenails and/or fingernails	N/A	No lunula involvement	Nail lacquer	Israel
Gupta, A. K. et al., 2005 [24]	Oral terbinafine & topical ciclopirox	Dermatophytes (not specified further)	Toenails	At least 1 great toenail	≥60% of the nail plate surface area and/or lunula/matrix involvement	Nail lacquer	Canada & US
Jaiswal, A. et al., 2007 [25]	Oral terbinafine & topical ciclopirox or amorolfine	*T. rubrum* (60%), *T. mentagrophytes* (13.3%), *T. tonsurans* (8.4%), *C. albicans* (15.6%), *Aspergillus* spp. (6.7%), *Scopulariopsis* spp. (2.2%)	Toenails and/or fingernails	N/A	N/A	Nail lacquer	India
Tietz, H. J. et al., 2013 [26]	Topical bifonazole & urea	*T. rubrum* (92–93%), *T. interdigitale* (5–6%), other (5–7%)	Toenails and/or fingernails	At least 1 but not more than 3 nails	Affected nail plate surface area 20–50% of target nail	Cream, paste	Germany
Bassiri-Jahromi et al., 2012 [27]	Topical fluconazole & urea	*T. rubrum* (78.8%), *T. mentagrophytes* (19.7%), *T. verrucosum* (1.5%)	Toenails and/or fingernails	N/A	At least 25% nail plate surface area of target nail and at least 2 mm of healthy nail from the nail fold to the proximal nail plate	Nail lacquer	Iran
Lecha, M. 2001 [28]	Oral itraconazole & topical amorolfine	*T. rubrum* (64.9%), *Candida* spp. (16.7%), *S. brevicaulis* (10.5%), *T. mentagrophytes* (8.8%), other (4.4%)	Toenails	At least 1 (not including little toenail)	Matrix area involvement and/or ≥80% total nail surface involvement	Nail lacquer	Spain
Rigopoulos, D. et al., 2003 [29]	Oral itraconazole & topical amorolfine	*C. albicans* (94.4%), *C. parapsilosis* (3.3%), other *Candida* spp. 2.2%)	Fingernails	N/A	At least 50% of the whole nail surface	Nail lacquer	Greece
Gupta, A. K. et al., 2001 [30]	Oral itraconazole & terbinafine	*T. rubrum* (92.1%), *T.* *mentagrophytes* (7.9%)	Toenails	N/A	N/A	N/A	Canada & US
Gupta, A. K. et al., 2013 [31]	Oral itraconazole & terbinafine	*T. rubrum* (86.7%), *T.* *mentagrophytes* (13.3%)	Toenails	At least 1 great toenail	20–100% affected nail plate surface area	N/A	Canada
Arenas, et al., 1991 [32]	Oral itraconazole or griseofulvin & topical isoconazole or urea	*T. rubrum* (37.7%), unknown (28.6%), mixed (16.6%), *Candida* spp. (16%), *T. mentagrophytes* (1.1%)	Toenails	At least 1 great toenail	N/A	Cream	Mexico
Zhang, J. et al., 2016 [33]	Laser & topical amorolfine	*T. rubrum* (88.9%), *C. albicans* (11.1%)	Bilateral fingernails and/or bilateral toenails	N/A	N/A	Nail lacquer	China
Bunyaratevej, S. et al., 2020 [34]	Laser & topical amorolfine	*N. dimidiatum* (75%), *Fusarium* spp. (25%)	Toenails	N/A	No involvement of nail matrix	Nail lacquer	Thailand
Zhang, J. et al., 2021 [35]	Laser & topical amorolfine	*T. rubrum* (67.9%), *Candida* spp. (19.2%), *T. mentagrophytes* (3.8%), *A. fumigatus* (1.3%)	Toenails	At least 1 great toenail	N/A	Nail lacquer	China
Bonhert, K. et al., 2019 [36]	Laser & topical efinaconazole	Dermatophyte or mixed dermatophyte/*Candida* spp.	Toenails	At least 1 great toenail	Uninfected length 3 mm or more (from the proximal nailfold) and 3 mm or less in thickness	Nail solution	US
Li, Y. et al., 2016 [37]	Laser & oral itraconazole	Not reported	Toenails and/or fingernails	N/A	N/A	N/A	China
Hamed Khater, M. & Khattab, F.M. 2020 [38]	Laser & oral itraconazole	Not reported	Toenails and/or fingernails	N/A	N/A	N/A	Egypt
Zaki, A.M. et al., 2020 [39]	Laser & topical tioconazole	Yeast (31%), non-dermatophytes molds (28.5%), dermatophyte (22%), *Trichosporon* spp. (18.5%)	Toenails and/or fingernails	N/A	N/A	Nail solution	Egypt
Zhou, B.R. et al., 2016 [40]	Laser & topical luliconazole	*T. rubrum* (74.8%), *T. mentagrophytes* (16.1%), *C. albicans* (9%)	Toenails and/or fingernails	N/A	N/A	Cream	China
Xu, Y. et al., 2014 [41]	Laser & oral terbinafine	Not reported	Toenails and/or fingernails	N/A	N/A	N/A	China
Kim, T.I. et al., 2016 [42]	Laser & topical naftifine HCl spray	*T. rubrum* (73.2%), *Candida* spp. (16.1%), *T. mentagrophytes* (10.7%)	Toenails and/or fingernails	N/A	N/A	Spray	Korea
Gilaberte, Y. et al., 2017 [43]	Methyl aminolevulinate photodynamic therapy & topical urea	*T. rubrum* (30%), *Aspergillus* spp. (15%), *T. mentagrophytes, Fusarium* spp. & other (all 7.5%), *S. brevicaulis* (5%)	Toenails and/or fingernails	N/A	N/A	Ointment	Spain
Jennings, M.B. et al., 2006 [44]	Debridement & oral terbinafine	Dermatophytes (not specified further)	Toenails	At least 1 great toenail	N/A	N/A	US
Potter, L.P. et al., 2007 [45]	Debridement & oral terbinafine	Not reported	Toenails	At least 1 great toenail	N/A	N/A	US
Malay, D.S. et al., 2009 [46]	Debridement & topical ciclopirox	*Candida* spp. (28%), *T. rubrum* & mixed (both. 23.2%), *Aspergillus* spp. & other saprophyte (both 10.4%), *T. mentagrophytes* (4.8%)	Toenails	N/A	N/A	Nail lacquer	US
Shemer, A. et al., 2016 [47]	Nail drilling, oral & topical terbinafine	*T. rubrum* (88.8%), *T. mentagrophytes* (11.2%)	Toenails	N/A	≤75% nail involvement with no lunula involvement	Spray	Israel

N/A: not applicable; US: United States.

## Data Availability

All data analyzed during this study are included in this article. Further enquiries can be directed to the corresponding author.
